# A Novel Approach to Estimate Mercury Exposure Risks Through Fish Consumption Based on the Selenium–Mercury Molar Ratio

**DOI:** 10.3390/toxics13080621

**Published:** 2025-07-25

**Authors:** Cássio da Silva Cabral, Lucas Cabrera Monteiro, Thiago Aluisio Maciel Pereira, Walkimar Aleixo da Costa Júnior, Iuri Aparecida da Silva Oliveira, Thayson Araujo Canela, José Vicente Elias Bernardi, Inácio Abreu Pestana, Ronaldo de Almeida

**Affiliations:** 1 Graduate Program in Regional Development and Environment, Federal University of Rondônia, Porto Velho 76801-059, Rondônia, Brazil; iuria.oliveira@gmail.com; 2 Wolfgang Christian Pfeiffer Environmental Biogeochemistry Laboratory, Federal University of Rondônia, Porto Velho 76801-059, Rondônia, Brazil; walkimar.costa@unir.br (W.A.d.C.J.); tac84454847@gmail.com (T.A.C.); ronaldoalmeida@unir.br (R.d.A.); 3 Graduate Program in Ecology, University of Brasília, Brasília 70910-900, Distrito Federal, Brazil; lcabreramonteiro@gmail.com; 4 Graduate Program in Chemistry, Chemistry Institute, University of Brasília, Brasília 70910-900, Distrito Federal, Brazil; thiagomaciel6198@gmail.com; 5 Geostatistics and Geodesy Laboratory, UnB Planaltina College, University of Brasilia, Planaltina 73345-010, Distrito Federal, Brazil; bernardi@unb.br; 6 Department of Geochemistry, Fluminense Federal University, Niterói 24020-141, Rio de Janeiro, Brazil; inaciopestana@id.uff.br

**Keywords:** Se:Hg ratio, biomagnification, risk biomarkers, toxicity, food safety

## Abstract

In contrast to mercury, an extremely toxic element, selenium is an essential micronutrient, which by complexing with mercury can mitigate its toxicity. In this regard, we quantified mercury and selenium concentrations in samples (n = 309) of fish tissues and analyzed the Se:Hg molar ratio and HBVSe as toxicological risk biomarkers. The data indicated that mercury levels in planktivorous fish (0.630 ± 0.202 mg kg^−1^) and carnivorous fish (1.196 ± 0.513 mg kg^−1^) were above the Brazilian limits considered safe for daily consumption. The highest selenium concentrations were observed in planktivores (0.272 ± 0.093 mg kg^−1^) and the lowest in herbivores (0.099 ± 0.092 mg kg^−1^). Molar ratios greater than one and positive HBVSe values were found in 42% of the fish samples (n = 131). As a result, we found that (i) the trophic level influences the risk of mercury exposure through the intake of fish in the diet; (ii) the approach presented in our study (model II) involves greater rigor concerning intake and exposure via fish consumption, since it considers the antagonistic Se:Hg ratio; and (iii) selenium can attenuate mercury toxicity, but safe thresholds vary depending on the species.

## 1. Introduction

Selenium (Se) is an essential element belonging to the chalcogen group of the periodic table [1]. Se is essential for the enzymatic activity of many proteins (selenoproteins) that act as antioxidants [2], with glutathione peroxidase (GSH-px) and selenoprotein P (SeIP) being the best known. Studies have identified 25 genes responsible for encoding selenoproteins in mammals [3,4]. Se levels can affect immunity as well as the expression of selenoproteins [5]. The limits of Se’s essentiality and toxicity are very tenuous. While the absence of Se in living organisms can cause physiological damage, chronic exposure leads to cases of intoxication [2,5,6].

In aquatic systems, Se chemical species are associated with three important processes: (i) exposure to selenate, selenite, elemental Se, and Se (-II) associated with deposition or resuspension; (ii) production of selenomethionine and selenocysteine, related to trophic transfer; and (iii) microbial activity, associated with the production of selenate, selenite elemental Se, and Se (-II) [7].

In the second half of last century, researchers observed that Se has the capacity to complex with metals such as mercury (Hg). This relationship results in insoluble HgSe complexes [8,9], as observed in studies of marine animals [10,11]. Some authors associated the presence of HgSe with a product of this relationship between the two elements, preventing the intoxicating action of Hg [8,10,12], since selenium can inhibit the accumulation of methylmercury (MeHg), induce Hg demethylation [13,14], and/or increase the elimination of methylmercury [14,15,16]. A Se:Hg molar ratio >1 is considered by many researchers to be an important factor of the potential protective action of Se [17,18,19,20]. However, Hg can manifest toxicity in the presence of Se deficiency [8], since Hg acts in the synthesis and incorporation of selenoproteins, impairing these processes [21]. It is essential to highlight that the protective effect of Se is a result of the antagonistic relationship between Se and Hg, which neutralizes and inhibits the toxic effects of Hg [22], as well as the detoxification process, which involves reducing toxicity and increasing the excretion of Hg [23].

The mitigation of Hg intoxication has been observed in studies with humans [23,24,25] and other mammals [20,26,27], seabirds [28,29], freshwater and marine fish [28,30,31,32,33], and seafood in general [34]. The possible protective effect of Se in relation to Hg may be an important factor for the health of people exposed to MeHg in the Amazon, since the average fish consumption in this region is 12 to 32 times greater than the Brazilian national average of 26 g day^−1^ [35,36,37]. Indeed, fish consumption is the main route of human exposure to Hg in the Amazon, where fish are important sources of protein and nutrients [38].

In geographically isolated communities, average consumption of up to 805 g day^−1^ was observed [37], while along the Madeira River, the main tributary on the right bank of the Amazon River, fish consumption varies from 320 to 406 g day^−1^ [36,39], although changes in eating habits have been observed in the lower Madeira River region [40,41,42]. Additionally, fish consumption increases with the geographic isolation of riverside communities from urban centers [38].

In the western Amazon, legal and illegal small-scale gold mining (ASGM) has been occurring for at least four decades. This gold is typically found in the form of small flakes in sediments, and metallic Hg (liquid phase) is used to amalgamate and separate the gold from these sediments [43]. Besides this activity, other anthropogenic sources are deforestation for agriculture and stock grazing, and construction of hydroelectric reservoirs, contributing to the remobilization and bioavailability of Hg and Se in aquatic ecosystems [44]. Especially in aquatic ecosystems, Hg undergoes chemical transformation to MeHg and bioaccumulates in the trophic chain until it reaches humans via fish consumption [45,46].

In the lower Madeira River, between the states of Rondônia and Amazonas, studies have highlighted the potential risks of fish consumption, especially of predatory fish species, in relation to Hg in riverine communities [36,40,41,47]. However, the classic toxicology of Minamata disease has not been reported in the region, even though the average Hg concentrations in more geographically isolated populations are higher than those recommended by Brazilian regulations [40,42]. On the other hand, recent studies have reinforced the protective role of selenium in relation to Hg toxicity [23,24,48,49,50]. Therefore, this study was guided by three main questions: (i) How do trophic levels influence selenium and total mercury concentrations in fish? (ii) Based on the Se:Hg molar ratio, health benefit value (HBVSe), and estimated daily intake (EDI), which fish species are safe for frequent human consumption? (iii) Can the traditional EDI model overestimate Hg intake when it disregards its interaction with Se? Based on these questions, our results provide a new and comprehensive framework for evaluating Hg exposure through fish consumption, considering the concurrent intake of Se.

## 2. Materials and Methods

### 2.1. Study Area

**Figure 1:**
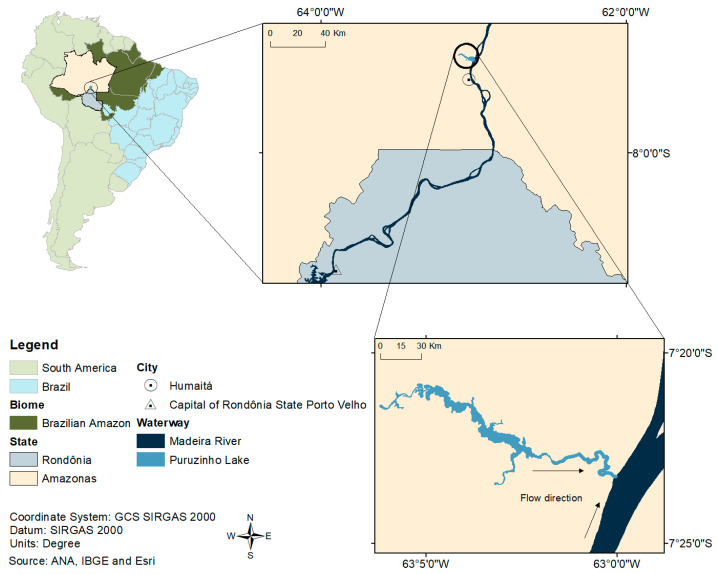
Map showing the location of the study area.

The study area was Puruzinho “Igarapé” (stream), which forms a lake with the same name, a tributary on the left bank of the Madeira River, located in the municipality of Humaitá, in the state of Amazonas (Figure 1). Lake Puruzinho is characterized by black water that seasonally is influenced by the clear water of the Madeira River (a phenomenon called “repiquete” in the region [51]. Its natural physicochemical characteristics are favorable to Hg methylation, present in high levels in biotic [51,52,53] and abiotic [51] environments. The land around the lake is the setting of a riverside community of the same name, whose residents live semi-isolated geographically and eat fish as their main source of animal protein. Around 123 individuals live there [42], potentially overexposed to Hg due to high fish consumption [36,41,53,54].

### 2.2. Sampling

Fish collection was carried out between December 2019 and November 2020, covering the four seasonal periods of the hydrological cycle in the Amazon region: rising water (RW), high water (HW), falling water (FW), and low water (LW). The fish samples were collected using nets with mesh between 30 and 70 mm. The individuals were identified to the species level and measured for total weight and standard length. An aliquot of the dorsal muscle was collected and stored in a freezer (−20 °C) until Hg and Se quantification. Fish species were selected based on local consumption and economic value in the Amazon region [36]. A total of 309 individuals from 17 species and 5 trophic guilds were collected: herbivores (n = 60), detritivores (n = 77), planktivores (n = 61), carnivores (n = 47), and piscivores (n = 64) (Table S1). The trophic guilds of each species were obtained from the FishBase platform [55]. The data on sample size, trophic level, habitat, total weight, and standard length are shown in Table S2.

### 2.3. Mercury Determination

To quantify total Hg (THg), samples of dorsal muscle weighing 200 mg (wet weight) were obtained from predatory fish and 400 mg from non-predatory fish. The samples were subjected to acid solubilization [56]. We added 0.5 mL of hydrogen peroxide (H_2_O_2_, 30%, Merck^®^, Rahway, NJ, USA), and after reaction, 4.0 mL of an acidic mixture (1:1, HNO_3_:H_2_SO_4_, Merck^®^). These samples remained for 30 min in a digester block (TE-007MP, Tecnal, Piracicaba, Brazil) at 70 °C. After the samples had cooled to room temperature, we added 5.0 mL of potassium permanganate (KMnO_4_ at 5% *w*/*v*, Merck^®^), again at 70 °C, for another 20 min in the digester block. The cooled samples were reserved overnight, and the next day the excess oxidant was removed with drops of hydroxylamine hydrochloride (NH_2_OH.HCl 12% *w*/*v*, Merck^®^). The samples were adjusted to a final volume of 14 mL with ultrapure water (Milli-Q, Millipore, Bedford, MA, USA). To determine THg, we applied atomic absorption spectrophotometry with cold vapor generation (CV-AAS, FIMS-400, PerkinElmer, Waltham, MA, USA).

### 2.4. Selenium Determination

To measure the selenium content, around 400 mg of dorsal muscle (wet weight) was used and acid solubilization was performed, according to Neubauer and Magarini (2021) [57]. About 0.5 mL of hydrogen peroxide (H_2_O_2_ 30%, Merck^®^) was added to each sample and then another 4.0 mL of 5.0 mol^−1^ nitric acid (HNO_3_ 65%, Merck^®^). After 30 min in the digester block at 85 °C and cooling to room temperature, 4.0 mL of hydrochloric acid (HCl 37%, Merck^®^) was added. Then after 30 more minutes in the digester block, the samples were filtered and transferred to falcon tubes, to which ultrapure water (Milli-Q) was added to obtain a final volume of 10.0 mL. Selenium determination was performed using inductively coupled plasma-optical emission spectrometry (ICP-OES, Optima 8300 PerkinElmer with hydride generation—HG).

### 2.5. Analytical Quality Control

Certified reference material was used in all analytical runs, and different techniques were used to guarantee the reliability of the analytical data. Certified tuna fish material (BCR-463, Joint Research Center) was used, with recovery of 97.7 ± 0.5% for THg (n = 8, LOD = 0.001 mg kg^−1^, and LOQ = 0.005 mg kg^−1^). For Se determination, dogfish muscle (DORM-2, National Research Council Canada) was used, with recovery of 102.5 ± 0.6% (n = 3, LOD = 0.005 mg kg^−1^, and LOQ = 0.015 mg kg^−1^). The glassware was previously decontaminated with HNO_3_ (3%, Merck ^®^), and analytical blanks were used to guarantee the purity of the reagents. All samples presented Hg and Se concentrations above their respective limits of detection (LOD).

### 2.6. Se:Hg Molar Ratio and Selenium Health Benefit Value

The molar ratio (Se:Hg) and the health benefit value of selenium (HBVSe) are based on the stoichiometry of the interaction between Se and Hg in organisms. Both indicators are based on the formation of stable molecular complexes of Se with Hg, limiting its bioavailability and interfering with cellular processes. Therefore, these indicators were calculated with the molarity of each element, expressing the number of atoms or molecules. The Se:Hg molar ratio was calculated using Equation (1) as proposed by Burger et al. [58], where Se is the concentration of selenium in mg kg^−1^ and 78.96 is the atomic mass of selenium, while Hg is the concentration of mercury in mg kg^−1^ and 200.59 is the atomic mass of Hg. A molar ratio greater than 1 indicates that Se is present in excess of Hg, and a ratio less than 1 indicates the opposite, with toxicological potential to impair physiological functions [18,59].

(1) $$Molar\;ratio=\frac{\left[molar\;Se\right]}{\left[molar\;Hg\right]}$$

The HBVSe is a risk assessment index in relation to fish consumption based on the protective effect of Se in relation to Hg [60]. HBVSe is an important indicator since it assesses the concentrations of Se and Hg in food and their impact on human health, since the concentration of Hg in food alone does not accurately indicate the risk of poisoning [60], so HBVSe is considered an appropriate and consolidated index in the literature. HBVSe was calculated using Equation (2), where Se is the molar concentration of selenium and Hg is the molar concentration of mercury in each sample [60]. Positive HBVSe values indicate that the individual has higher concentrations of selenium compared to mercury, suggesting the protective effect of selenium. In contrast, negative HBVSe values indicate higher concentrations of mercury compared to selenium, so there is no neutralization of the toxic effects of Hg by Se.

(2) $$HBVSe=(\left[Se-Hg\right])/Se)\times (Se+Hg)$$

### 2.7. Estimates of Hg Intake Through Fish Consumption

The Hg concentrations in all samples (n = 309) were compared directly with the limits defined for carnivorous and non-carnivorous fish by Brazilian regulations [61], which is the same as that used by international organizations [62]. Next, we used two models to estimate daily Hg intake through fish consumption using distinct approaches for comparison purposes. We select a subset of samples representative of all trophic guilds, aiming to select only samples with Se:Hg molar ratios lower than zero (i.e., excess concentrations of Hg relative to Se) (n = 178, Table S3).

In the first model, we used the traditional estimated daily intake (EDI), calculated by multiplying the total Hg concentrations determined in each fish sample by the fish intake rate (from different trophic guilds) for the Amazon and then dividing by the average weight of the human population (model I). The Hg concentrations were obtained in our study, fish consumption data (406 g day^−1^) were extracted from a previous study in the Puruzinho Lake [36], and population weight data (65 kg) were obtained from the Brazilian Institute of Geography and Statistics (IBGE) [63].

In the new approach to calculate EDI presented in this study (model II), in addition to total Hg concentrations, fish intake, and population weight, the concomitant intake of Se was also considered. This is a novel approach that considers the protective effects of Se. Here, we assumed that all ingested Se would have a protective effect against Hg, by forming SeHg, rendering Hg unavailable to cause harm to the organism. In this sense, we calculated the risk of Hg ingestion considering only the excess concentrations of Hg relative to Se (Hg_Free_). Molar concentrations of Se were subtracted from those of Hg (expressed in molar units), and the resulting Hg concentrations were then multiplied by its atomic weight (200.59) to convert values to mass-based units (mg kg^−1^, wet weight). After this calculation, all computations were performed as in model I. The limits were compared with those defined by the model of the Joint FAO/WHO Expert Committee on Food Additives (JECFA) [62], which establishes the provisional upper tolerable weekly intakes of Hg (PTWI limit) of 1.6 µg kg week^−1^ (or 0.57 µg kg day^−1^). Considering that PTWI values are based on MeHg concentration, we used THg as a proxy for MeHg [40].

### 2.8. Hg and Se Modeling via Fuzzy Logic

Fuzzy logic was employed to develop an integrated risk–benefit indicator for mercury exposure through fish consumption. Total Hg concentrations and the corresponding estimated daily intake (EDI, model II) were treated as risk indicators, with higher values indicating greater risk. In contrast, the molar ratio (Se:Hg) and the health benefit value of selenium (HBVSe) were considered benefit indicators, where higher values reflect greater protective effects. The integrated risk–benefit indicators were derived from the cost-efficiency framework proposed by Campos et al. [64].

The variables were classified into five risk levels (Table 1). Total Hg, Se:Hg, and HBVSe were stratified based on the 5th, 25th, 50th, 75th, and 95th percentiles, while estimated daily intake (EDI) values were categorized according to regulatory safety thresholds (Table 1). Harmony degrees (HDs) were then calculated for both risk and benefit indicators, standardizing the outputs on a 0–1 scale (see Luo et al. [65] for methodological details). Equal weights were assigned to each variable, and the integrated risk–benefit index was calculated using Equation (3), incorporating the protective role of selenium. The final results were normalized by data range, producing a risk scale for each fish sample: very low (0–0.20), low (0.21–0.40), moderate (0.41–0.60), high (0.61–0.80), and very high (0.81–1.00).

(3) $$Risk-Benefit\;Index\;=\;\left(HD_{Hg}+\;HD_{EDI_{Hg}}\right)-\left(HD_{Se:Hg}+\;HD_{HBV_{Se}}\right)$$

### 2.9. Statistical Analysis

We applied the Shapiro–Wilk test (for n < 50) and the Kolmogorov–Smirnov test (for n > 50) to assess data distribution. Differences in THg, Se, Se:Hg, and HBVSe were evaluated using the Kruskal–Wallis test, followed by Dunn’s post hoc test (n = 309). The integrated risk–benefit index showed a normal distribution; therefore, inter-guild comparisons were performed using analysis of variance (ANOVA) followed by Tukey’s post hoc test (n = 178). Kendall’s non-parametric correlation was employed to assess the relationships between THg and Se concentrations and fish standard length and weight. Although some correlations were statistically significant within certain trophic guilds, the strength of these associations was weak to moderate (Table S4). Therefore, THg and Se concentrations were not adjusted for body size prior to the comparative analyses. Paired t-tests and Wilcoxon signed-rank tests were used to compare THg and Se concentrations within the same samples from each trophic guild, as well as to compare intake estimates from the two models (traditional and Se-inclusive) (n = 178). A significance level of *p* < 0.05 was adopted for all statistical analyses.

Principal component analysis (PCA) was used to evaluate the dissimilarity of THg and Se concentrations, standard length and weight of fish, considering the most abundant species of the five trophic guilds evaluated, namely *Mylossoma aureum* (herbivore, n = 29), *Hemiodus unimaculatus* (detritivore, n = 34), *Hypophthalmus marginatus* (planktivore, n = 37), *Cichla pleiozona* (carnivore, n = 36), and *Serrasalmus rhombeus* (piscivore, n = 38) (Table S5). All comparison tests were carried out using Prism^®^ 10.4.1 (GraphPad Software, San Diego, CA, USA. License: Federal I.D. #33-0386987). Principal component analysis was performed in the R programming environment [66].

## 3. Results

### 3.1. Influence of Trophic Guild on Mercury and Selenium Concentrations

**Figure 2:**
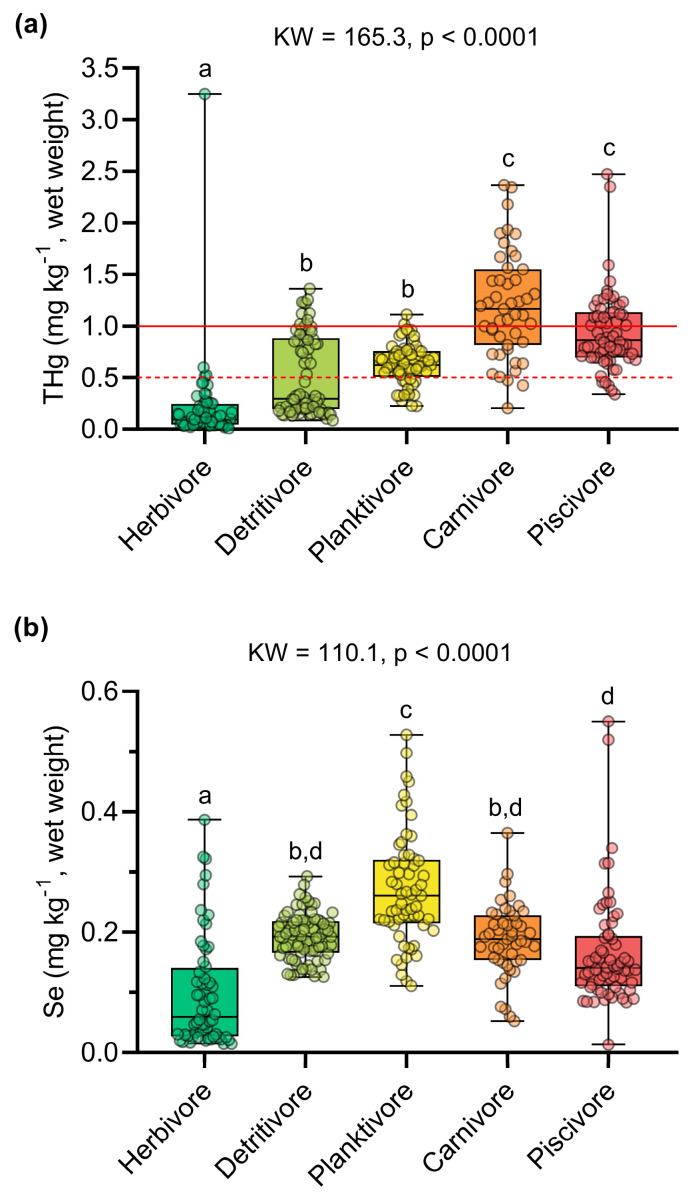
Comparison of the concentrations of (**a**) total mercury (THg) and (**b**) selenium (Se) between the trophic guilds. The letters above the boxplots indicate significant differences determined by Dunn’s post hoc test (*p* < 0.05). In (**a**), the solid and dashed red lines indicate the Hg safety limits for predatory species (1 mg kg^−1^) and non-predatory species (0.5 mg kg^−1^), respectively [61].

THg concentrations were significantly higher than Se concentrations in all trophic guilds (*p* < 0.05, Table S6). Hg concentrations were above those recommended by Brazilian regulations in 44% of the samples (n = 136). At the species level, average THg concentrations above the Brazilian safety limit were determined for the predatory species *Calophysus macropterus*, *Cichla pleiozona*, and *Pellona castelnaeana* (>1 mg kg^−1^), and the non-predatory species *Anodus elongatus*, *Hemiodus unimaculatus*, and *Hypophtalmus marginatus* (>0.5 mg kg^−1^) [67]. Carnivorous (1.196 ± 0.513 mg kg^−1^) and piscivorous (0.947 ± 0.382 mg kg^−1^) species had the highest THg concentrations, followed by planktivorous (0.630 ± 0.202 mg kg^−1^), detritivorous (0.517 ± 0.382 mg kg^−1^), and herbivorous (0.205 ± 0.425 mg kg^−1^) species (KW = 161.4; *p* < 0.0001) (Figure 2a, Table S7).

**Figure 3:**
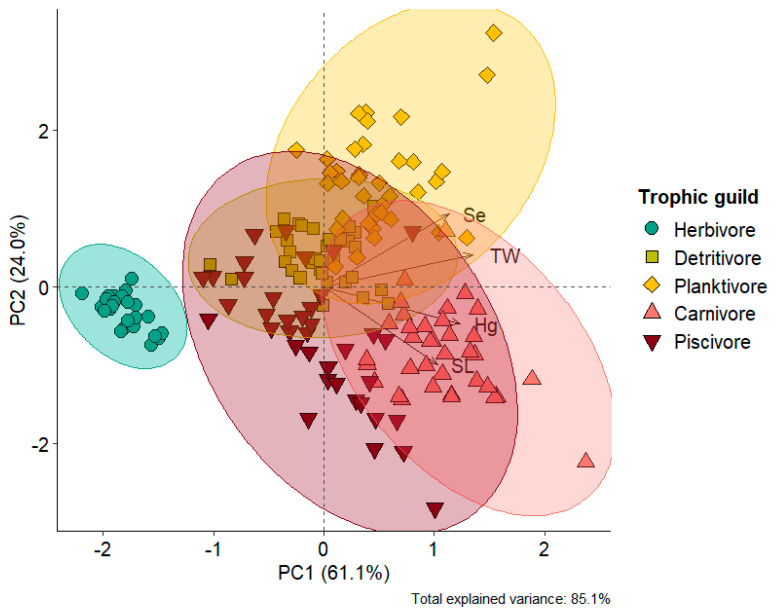
Bivariate plot of the projection of eigenvectors and scores on the principal axes (eigenvalues) of the principal component analysis, including Hg (mg kg^−1^) and Se (mg kg^−1^) concentrations, standard length (SL), and total weight (TW) in representative species from the five guilds, namely *Mylossoma aureum* (herbivore), *Hemiodus unimaculatus* (detritivore), *Hypophthalmus marginatus* (planktivore), *Cichla pleiozona* (carnivore), and *Serrasalmus rhombeus* (piscivore).

Selenium concentrations also varied among trophic guilds (KW = 110.1; *p* < 0.0001), exhibiting different bioaccumulation patterns compared to mercury. The highest concentrations were found in planktivorous species (0.272 ± 0.093 mg kg^−1^) and detritivorous species (0.193 ± 0.038 mg kg^−1^). In contrast, the lowest Se concentrations were observed in herbivorous (0.099 ± 0.092 mg kg^−1^), piscivorous (0.166 ± 0.091 mg kg^−1^), and carnivorous (0.188 ± 0.060 mg kg^−1^) species (Figure 2b, Table S7). Principal component analysis explained 85.1% of the variation in the data. Axis 1 (61.1%) revealed a high dissimilarity of the herbivorous species *Mylossoma aureum* in relation to the other trophic guilds, which were inversely ordered according to THg and Se concentrations (Figure 3). Indeed, the herbivore guild exhibited the lowest THg and Se concentrations in our dataset (Figure 2). Axis 2 (24.0%) showed a clear separation between the predatory species *C. pleiozona* and *S. rhombeus* (carnivorous and piscivorous, respectively), associated mainly with THg concentrations and the weight of individuals. In turn, the planktivorous species *H. marginatus* was associated with high Se concentrations and the length of individuals. The detritivorous species *H. unimaculatus* had a large overlap with the piscivorous and planktivorous species (Figure 3).

### 3.2. Se:Hg Molar Ratios and Selenium Health Benefit Value (HBVSe)

**Figure 4:**
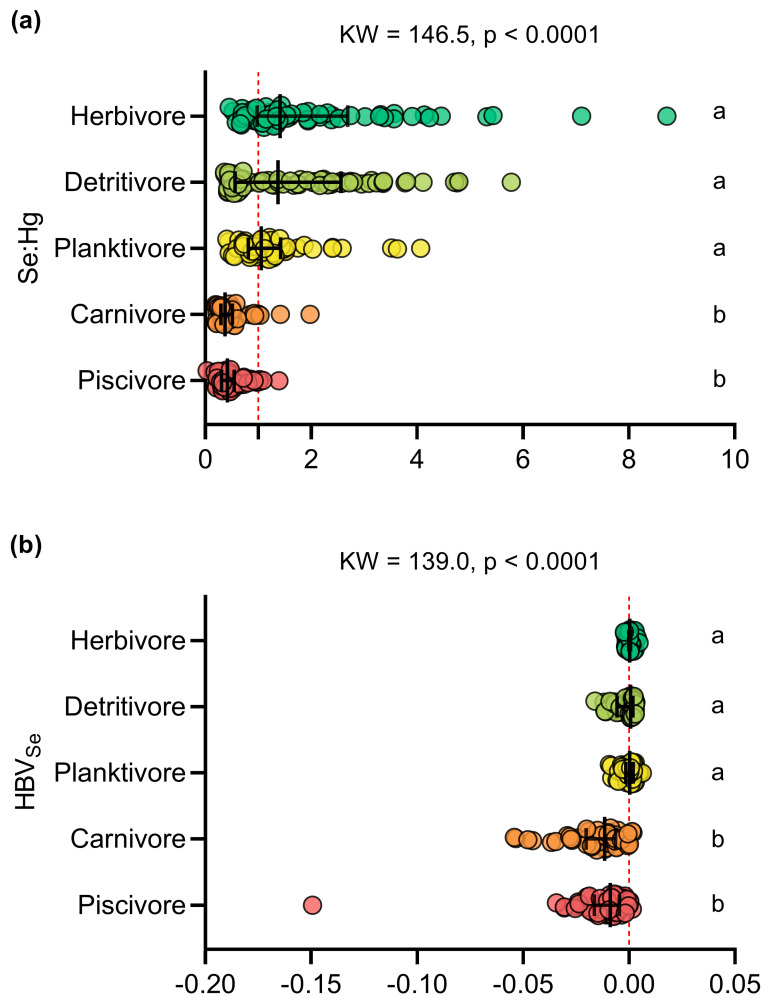
Comparison of the (**a**) selenium–mercury molar ratio (Se:Hg) and (**b**) selenium health benefit value (HBVSe) between the trophic guilds. The letters indicate significant differences determined by Dunn’s post hoc test (*p* < 0.05).

Molar ratios greater than one and positive HBVSe values were found in 42% of the fish samples (n = 131), with a predominance of detritivorous (n = 45), herbivorous (n = 45) and planktivorous species (n = 34), along with a small number of carnivores (n = 3) and piscivores (n = 4). The herbivorous (2.085 ± 1.646), detritivorous (1.706 ± 1.304), and planktivorous (1.246 ± 0.735) species had Se:Hg molar ratios significantly higher than those determined for carnivorous (0.485 ± 0.337) and piscivorous (0.484 ± 0.258) species (KW = 146.5; *p* < 0.0001) (Figure 4a, Table S7). Similarly, HBVSe values were significantly higher in herbivorous species (0.0005 ± 0.0013), followed by planktivores (−0.00005 ± 0.0032) and detritivores (−0.0017 ± 0.0047), and finally, piscivores (−0.0126 ± 0.0193) and carnivores (−0.0157 ± 0.0142) (KW = 139; *p* < 0.0001) (Figure 4b, Table S7).

### 3.3. Estimated Daily Intake of Hg Through Fish Consumption

The estimated daily intake (EDI) of Hg based on the traditional approach (model I) ranged from 1.71 ± 1.22 µg kg day^−1^ for herbivores to 7.78 ± 3.05 µg kg day^−1^ for carnivores (Table 2). All trophic guilds showed average Hg consumption above that recommended by the FAO (0.57 µg kg day^−1^) [67]. However, the estimates of Hg intake by detritivorous fish were significantly lower than those of the other guilds, with EDI below the limit for four individuals of the *M. aureum* species.

Based on the concomitant ingestion of Se (model II), all trophic guilds showed significant reductions in estimated Hg intake compared to the traditional approach (model I) (*p* < 0.05, Table S6). Mean Hg intake ranged from 0.43 ± 0.36 to 4.86 ± 2.93 µg kg^−1^ day^−1^ for herbivores and carnivores, respectively (Table 2). Despite the reduction in estimated intake, only the herbivore trophic guild showed a mean Hg intake below the regulatory threshold of 0.57 µg kg^−1^ day^−1^, representing a 73% reduction compared to model I. The smallest decreases in EDI between models were observed for carnivorous (41.8 ± 19.4%) and piscivorous (44.1 ± 19.8%) fish (Table 2). Under model II, approximately 15% of the samples (n = 26) exhibited EDI values below the safety threshold, mainly herbivorous (n = 10) and planktivorous (n = 10) species, with lower occurrences among carnivorous (n = 3) and piscivorous species (n = 3).

### 3.4. Integrated Risk–Benefit Index

**Figure 5:**
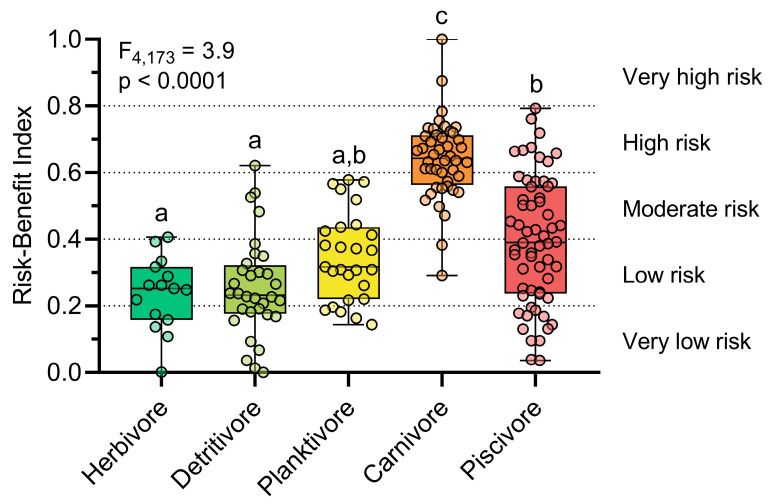
Comparison of the risk–benefit index, based on fuzzy logic and harmony degrees, between the trophic guilds. The letters indicate significant differences determined by Dunn’s post hoc test (*p* < 0.05). Risk levels: very low (0–0.2), low (0.21–0.4), moderate (0.41–0.6), high (0.61–0.8), and very high (0.81–1).

The integration of THg, EDI (model II), Se:Hg, and HBVSe using harmony degrees and fuzzy logic revealed that most fish samples posed a low (33.7%) or moderate (24.7%) consumption risk, followed by high (21.9%) and very low (18.5%) risk levels. Only two individuals of the carnivorous species *Cichla pleiozona* exhibited a very high consumption risk (1.1%) (Figure 5, Table S8). In addition, over half of the individuals who presented a high risk were from the *C. pleiozona species*. Therefore, *C. pleiozona* presents the highest risk of consumption among the species evaluated in our study. The safest species for consumption was the detritivore *Hemiodus unimaculatus*, which showed the highest proportion of samples with low and very low risk. The planktivorous species *Hypophthalmus marginatus* and the piscivorous *Serrasalmus rhombeus*, in turn, were predominantly associated with low to moderate risk levels.

In addition to the variation in risk between species, the distributions of risk levels varied substantially between trophic groups (F_4,173_ = 38.87, *p* < 0.0001) (Figure 5, Table S8). Herbivores and detritivores presented mostly low risk (60.0% and 53.1%, respectively), followed by very low risk (33.3% and 34.4%, respectively). Planktivores had a higher frequency of low risk (48.1%) and moderate risk (33.3%). In contrast, carnivores were predominant in the highest-risk categories: 65.9% of individuals were classified as high risk and 25.0% as moderate, with only 4.5% at low risk. Piscivores showed a wider distribution, with 33.3% at moderate, 31.7% at low, and 20.0% at very low risk, while 15.0% were classified as high and 4.5% as very high risk. These results indicate a gradient of increasing risk along the trophic chain, with carnivores and piscivores concentrating the highest proportions in the high-risk classes.

## 4. Discussion

### 4.1. Biomagnification of Total Mercury (THg) in the Food Chain

Lakes are depositional environments that receive Hg from natural and anthropogenic sources, where the hydrodynamic properties, such as the occurrence of anoxic zones and the high content of organic matter, favor the bioaccumulation and biomagnification of Hg in abiotic and biotic compartments [68]. This process is intensified in floodplains, where seasonal dynamics alter the physicochemical conditions (pH and dissolved oxygen), depth, and content of dissolved organic matter in lakes [68,69,70,71]. Hence, relatively high concentrations of Hg were observed in the bottom sediment (0.03 and 0.15 mg kg^−1^; [51]) and in the planktonic community (0.03 and 0.34 mg kg^−1^; [72]) of Lake Puruzinho, representing important sources of Hg for fish.

Our data show that planktivorous, carnivorous, and piscivorous fish had concentrations above the recommended limit for human consumption according to Brazilian legislation [61] (Figure 2a), in accordance with previous studies carried out in Lake Puruzinho [36,41,53,73,74]. Total Hg concentrations in Amazonian aquatic and terrestrial plants vary between 0.02 and 0.06 mg kg^−1^ [71,75], justifying the lower concentrations in herbivorous fish. In contrast, despite the high concentrations in the bottom sediments of Lake Puruzinho and the suspended particulate matter of the Madeira River basin (0.05–2.62 mg kg^−1^; [76]), the low availability of organic Hg in these matrices reduces dietary exposure in detritivorous fish [72,77].

Land use changes can also modify limnological conditions and intensify the transport of inorganic Hg bound to soil particles into aquatic ecosystems, altering the distribution at the base of trophic chains and in the ichthyofauna [78,79,80,81]. Lacerda et al. (2024) [44] found a temporal increase in Hg concentrations in the detritivorous species *Prochilodus nigricans* and the carnivorous species *Cichla pleiozona,* with higher concentrations in the lakes compared to the main channel of the Madeira River in the most recent years (2019–2021). This temporal trend was mainly attributed to intense deforestation in the Madeira River basin [44]. Indeed, Almeida et al. (2014) [51] showed that the spatial distribution of Hg in sediments is affected by the lateral transport of water from the Madeira River during the flood period. Additionally, despite the reduction in mining activity in recent years, artisanal and small-scale gold mining is an important factor for the high Hg concentrations in fish in the Amazon [82].

### 4.2. Patterns of Selenium (Se) Bioaccumulation Among Trophic Guilds

Selenium concentrations in fish from Lake Puruzinho were lower than those found by Dorea et al. (1998) [33] in the Madeira River basin (state of Rondônia, Brazil) and Lima et al. [32] in the state of Pará (Brazil) and are closer to those found by Albuquerque et al. [83] in Santarém (state of Pará, Brazil) and by Lino et al. [77] and Sampaio da Silva et al. [84] in the Lower Tapajós (state of Pará). The relatively small number of studies on Se in Amazonian fish makes it difficult to determine clear patterns of bioamplification. For example, Dorea et al. (1998) [33] demonstrated that Se concentrations increase according to trophic level, while Lino et al. [77] indicated higher Se concentrations in fish from low trophic levels. In our study, the highest concentrations of Se were found in planktivorous fish, which occupy intermediate trophic levels in fish communities.

Despite the low concentrations of Se in the herbivore trophic guild in our study, there was no clear pattern of bioamplification in the trophic chain. Detritivorous species, which occupy the base of the trophic chain, showed similar concentrations to carnivorous species. This behavior may be related to the low availability of Se in the environment [85], although the biomagnification of Se in trophic guilds does not have a pattern or behavior established in the literature [86]. It should be noted that freshwater fish tend to contain lower concentrations of Se than marine fish [86]; however, most of the Se present in freshwater fish is in organic form (selenomethionine), which can increase the excretion of Hg and hence reduce its possible toxicological effects [87]. In addition, fish may increase their absorption of Se in response to exposure to Hg, as a way of ensuring the production of selenoproteins and mitigating Hg toxicity [88], justifying the inverse pattern of THg and Se across trophic guilds shown by PCA.

The heterogeneity of the distribution and bioavailability of Se in the soil and aquatic environment is complex and associated with various factors such as physicochemical parameters, organic matter, and chemical speciation [89]. The soil in the study area has a mean Se concentration of 0.355 mg kg^−1^ (unpublished data), below the global average (0.400 mg kg^−1^) [90,91] and Se-deficient (<0.500 mg kg^−1^) [92]. Geographical location, anthropogenic sources, atmospheric deposition, and geological formations all influence Se levels in the environment [93]. In our study area, deep oxisols (>2 m) with low fertility and high acidity predominate [94], reducing the availability of Se.

Se concentrations in plants tend to reflect the concentrations of this element in the soil [91,95]. In addition, soil acidity is a crucial factor in the absorption of Se by plants, as it facilitates the formation of insoluble HgSe compounds under slightly acidic pH conditions, thereby limiting its absorption by plants [95,96,97]. Even soils rich in Se and organic matter can have the bioavailability of this micronutrient affected by pH [91]. The relationship between soil acidity and Se content was observed in the Amazon in Bertholletia excelsa (Brazil nut), showing that more acidic soils produced fruit with lower Se contents than slightly alkaline soils [89,98]. This is most likely because acidic soils tend to decrease the bioavailability of Se (IV), the most soluble and bioavailable form of Se in the soil [99]. Given that the diet is the main route of Se absorption for living organisms [6], the low levels of Se in herbivorous fish may reflect the low levels of Se in the soil and the low capacity of most plants to absorb Se.

### 4.3. Health Benefit Value of Se and Estimates of THg Intake by the Riverside Population

The selenium health benefit value (HBVSe) was mostly negative in our dataset (Figure 4b), different from the values found in freshwater fish from Brazil [78] and other regions of the world [93,100]. However, the higher HBVSe values in non-carnivorous species are consistent in the scientific literature. In the Brazilian Amazon, Lino et al. [77] obtained negative HBVSe values only for carnivorous species, including the voracious predators of the Cichla genus. In northern Colombia, negative HBVSe values were obtained for opportunistic omnivorous and carnivorous species, which have a wide variety of food sources and, consequently, greater exposure to Hg through the diet [93].

Along these lines, the Se:Hg molar ratio smaller than one in predators and the negative HBVSe values can be explained by the lack of physiological functions of Hg [46] and its relatively high concentrations in the biotic and abiotic compartments of our study area [41,51,52,72,74,101], intensifying the bioaccumulation and biomagnification of Hg in fish. It is necessary to note that the Hg concentrations in fish from the Madeira River are higher than those determined in the studies mentioned above [77,93,100], justified mainly by the ASGM and the intensification of deforestation in recent decades, as previously discussed [43,44,102].

In contrast, Se is an essential chemical element for metabolic reactions and has homeostatic control in organisms, thus controlling the concentrations of this element, regardless of the concentrations in the environment and according to physiological needs [103]. In addition, the low capacity for bioaccumulation and biotransfer in the trophic chain of Se compared to Hg (Figure 2b) directly implies the results of the molar ratio and benefit value. Recently, Pontes et al. (2025) [104] showed that higher concentrations of Se in the blood can mitigate the metabolic changes induced by Hg in humans. However, only the Se available in fish is not sufficient to mitigate the toxic effects of Hg in our study area. For example, Hg concentrations were higher than Se concentrations in all the species analyzed in our study, while Córdoba-Tovar et al. (2025) [93] determined Se concentrations twice as high as Hg concentrations in carnivorous and non-carnivorous species, resulting in molar ratios predominantly greater than one.

A large cohort study conducted in the Brazilian Amazon indicated that Hg concentrations in the population’s blood increased according to fish consumption; however, no relationship was observed between fish consumption and Se concentration [105]. Similarly, positive correlations between the consumption of carnivorous fish and Hg concentrations in hair were described in a riverside community along the Tapajós River (Amazon), but no significant relationship with the consumption of non-carnivorous fish was observed [106]. In addition to the comparatively low Hg concentrations, low-trophic-level species have high nutritional value, high abundance in Amazonian ecosystems, and low commercial value, thus representing an important food source for riverine populations [107]. Supplementing the diet with other sources of Se, including Brazil nuts and fruits, is also an important factor increasing Se levels in the body and mitigating the toxicological effects of Hg [107,108]. In addition to selenium’s protective effect on Hg toxicity, fruit consumption alters the kinetics of Hg in the human organism [106,109]. In an epidemiological study in the Tapajós River region, ref. [110] observed an inverse relationship between Hg concentrations and fruit consumption, attributed to the content of soluble dietary fiber and probiotic nutrients in fruits that could affect absorption in the gastrointestinal tract.

The concentrations of Hg in herbivorous and detritivorous species were considered safe for human consumption according to Brazilian regulations [61]. However, the safe limit does not consider the high fish consumption typical of riverine residents in the Amazon. Despite the dietary diversification promoted by the nutritional transition in recent decades, fish is still the main source of protein for these people [110,111,112], causing greater vulnerability to Hg exposure [113]. The herbivores *Mylossoma* ssp. are the main source of fish protein in the neighboring riverside community Demarcação, with estimated daily consumption within the limits established by the FAO [40]. However, the fish consumption estimated by Canela et al. [40], based on the average consumption of riverside communities on the Madeira River [39], was 20% lower (320 g day^−1^) than those reported in our study area (406 g day^−1^) [36]. In addition, people living near Lake Puruzinho consume fish on average four to five times a week [42], intensifying dietary exposure to Hg.

When considering only the fish consumption (model I), the estimates of daily Hg intake exceeded the provisional tolerable limit (0.57 µg kg day^−1^) set by the Joint FAO/WHO Expert Committee on Food Additives (JECFA) [62]. Using the new approach proposed here (model II), based on consumption rate and Se-free Hg concentration, only fish of the herbivore guild were considered safe for consumption. It is important to note that, according to model II, the average intake estimates were substantially reduced, showing a reduction of between 41.8 and 44.1% for predatory species and between 54.3 and 75.7% for non-predatory species (Table 2). Thus, by only considering Hg concentrations, the traditional model could potentially overestimate Hg intake estimates through fish consumption.

The integrated assessment of Hg and Se intake, considering both risk indicators (THg and EDI) and benefit indicators (Se:Hg and HBVSe), confirmed that herbivorous and detritivorous fish present a lower risk of Hg exposure. This finding is particularly relevant since the local population in our study area primarily consumes herbivorous and detritivorous fish [36]. Although the HBVSe values in these trophic guilds were relatively modest, they may help mitigate health risks. Positive HBVSe in food is important because it reflects the availability of Se for the synthesis of selenoproteins and the enhancement of enzymatic activities such as thioredoxin reductase (TrxR) [114]. Selenoproteins play a crucial role in counteracting Hg toxicity by reducing oxidative damage (via glutathione peroxidase, GPx), transporting Se from plasma to the brain (selenoprotein P, SelP) [115,116], and regulating the redox state (TrxR), thus maintaining selenoprotein activity. Essentially, selenoproteins detoxify Hg and mitigate Hg-induced oxidative stress. In this context, modeling exposure risk using fuzzy logic that incorporates the protective effects of Se enabled the identification of patterns that might be overlooked when evaluating Hg concentrations alone. For example, although Hg biomagnification along the trophic chain is evident, with higher concentrations in piscivorous and carnivorous fish (Figure 2), the integrated risk–benefit index indicated that the piscivorous species *Serrasalmus rhombeus* may pose a very low to moderate risk to human consumers (Table S8).

The five-level risk gradient enabled the identification of the safest species for consumption. In addition to *Serrasalmus rhombeus*, the detritivorous species *Hemiodus unimaculatus* is recommended, as it consistently exhibited low to very low risk levels. The planktivorous species *Hypophthalmus marginatus* showed low to moderate risk, suggesting it should be consumed with caution. Conversely, the carnivorous species *Cichla pleiozona* predominantly presented high to very high risk levels and should be avoided when possible. Thus, the proposed framework, which integrates fuzzy logic and harmony degrees within a risk–benefit approach, provides a realistic and practical tool to enhance the accuracy of risk assessments in fish-dependent populations. Beyond its ease of interpretation by both the population and decision-makers, a key strength of this approach lies in its flexibility to incorporate diverse numerical variables and assign weights proportional to their respective risk or benefit of Hg and Se intake.

## 5. Conclusions

Total Hg concentrations above those recommended by Brazilian regulations were determined for predatory species (>1 mg kg^−1^; n = 56) and non-predatory species (>0.5 mg kg^−1^; n = 81). Hg concentrations were significantly higher than Se concentrations and showed different bioaccumulation patterns among the trophic guilds. Hg concentrations had a clear pattern of biomagnification in the trophic chain, while Se concentrations did not differ between carnivorous and non-carnivorous species. The lowest concentrations of both elements were found in herbivorous fish, showing greater dissimilarity with the other trophic guilds.

We found molar ratios greater than one and positive HBVSe values in 42% of the fish samples (n = 131), with a predominance of detritivorous, herbivorous, and planktivorous species. However, our results showed that the Se concentrations were not sufficient to reduce Hg exposure. Despite the comparatively lower estimated intake of species with low trophic level, daily intake exceeded the safe limit, except for the herbivore guild in the model considering Se-Hg antagonism (new approach). According to the traditional approach to estimate intake, only four individuals of the herbivorous species *Mylossoma aureum* had intake below the safe limit. In contrast, according to the new approach proposed here, 26 individuals had estimated intake below the safe limit, including predatory species.

Brazilian regulations on safe levels of Hg for human consumption only consider the absolute concentration in fish, underestimating the risk of exposure of people for whom fish is the main source of protein. Furthermore, the traditional approach to estimating daily intake (model I) considers fish consumption and total Hg concentrations, including those complexed with Se, potentially overestimating the intake rate. The approach presented here (model II) proposes more stringent limits concerning actual intake and exposure via fish consumption, since it considers the Se-Hg relationship. It is important to note that this approach is only functional for samples whose Hg molarity exceeds that of Se. It can be widely applied in neotropical ecosystems (especially in the Amazon) and regions impacted by anthropogenic sources of Hg.

The application of fuzzy logic and harmony degrees to create an integrated risk–benefit index provides a novel framework for interpreting commonly used metrics in the scientific literature (e.g., Se:Hg and HBVSe). Fuzzy logic-based indices have been applied in the environmental management context, delivering results that are easily interpreted by both the scientific community and decision-makers [64,65,117]. Here, we classified fish under different gradients of Hg and Se concentrations into five risk categories, making it possible to identify the safest species (*Hemiodus unimaculatus* and *Serrasalmus rhombeus*) for consumption and those that should be avoided when possible (*Cichla pleiozona*). In this context, we emphasize that our study is strictly in accordance with Article 19 of the Minamata Convention, which highlights the importance of geographically representative modeling and monitoring of Hg concentrations in vulnerable populations and in the environment (including in fish) [118].

## Figures and Tables

**Table 1:** Classification of input variables for normalization by harmony degrees and calculation of the risk index. Risk indicators: the higher the value, the greater the risk. Benefit indicators: the higher the value, the greater the benefit.

Indicator	Class	Unit	Excellent	Good	Moderate	Poor	Very Poor
Hg	Risk	mg kg^−1^	0.04	0.7	1.0	1.5	2.5
EDI—Model II	Risk	µg kg day^−1^	0.57	2	5	8	12
Se:Hg	Benefit	unitless	0.977	0.735	0.564	0.465	0.279
HBVSe	Benefit	unitless	−0.0006	−0.0030	−0.0063	−0.0108	−0.0342

**Table 2:** Descriptive statistics of the estimated daily intake (EDI) of Hg using the traditional approach (model I) and considering the concomitant intake of Se (model II) (µg kg day^−1^). The reduction in EDI between the models is indicated as a percentage (%). SD: standard deviation. IQR: interquartile range. Range: minimum to maximum. The EDI was calculated based on the consumption of fish by the local population (406 g day^−1^) and the average body weight of the adult population of the state of Amazonas (65 kg).

Trophic Guild	EDI—Model I (µg kg day^−1^)			EDI—Model II (µg kg day^−1^)			Reduction (%)
	Mean (SD)	Median (IQR)	Range	Mean (SD)	Median (IQR)	Range	
Herbivorous (n = 15)	1.71 (1.22)	2.01 (2.37)	0.27–3.47	0.43 (0.36)	0.30 (0.68)	0.03–1.07	73.3 ± 14.6
Detritivorous (n = 32)	5.85 (1.24)	5.84 (1.39)	2.87–8.49	2.75 (1.05)	2.67 (1.38)	0.59–5.38	54.3 ± 11.1
Planktivorous (n = 27)	4.55 (1.20)	4.50 (5.63)	2.10–6.93	1.21 (1.02)	0.92 (1.58)	0.11–3.53	75.7 ± 17.1
Carnivorous (n = 44)	7.78 (3.05)	7.54 (4.09)	2.65–14.76	4. 86 (2.93)	4.27 (3.18)	0.02–11.68	41.8 ± 19.4
Piscivorous (n = 60)	6.04 (2.33)	5.49 (2.62)	2.13–15.44	3.53 (2.02)	3.16 (2.44)	0.16–10.46	44.1 ± 19.8

## Data Availability

The data presented in this study are available from the corresponding author upon request.

## Supplementary Materials

The following supporting information can be downloaded at: https://www.mdpi.com/article/10.3390/toxics13080621/s1, Table S1: Raw data; Table S2: Sample characterization; Table S3: Estimated daily intakes—model I and model II; Table S4: Kendall correlation; Table S5: Principal component analysis; Table S6: Paired comparisons; Table S7: Multiple comparisons; Table S8: Risk–benefit index.
